# Deep learning models of ultrasonography significantly improved the differential diagnosis performance for superficial soft-tissue masses: a retrospective multicenter study

**DOI:** 10.1186/s12916-023-03099-9

**Published:** 2023-10-26

**Authors:** Bin Long, Haoyan Zhang, Han Zhang, Wen Chen, Yang Sun, Rui Tang, Yuxuan Lin, Qiang Fu, Xin Yang, Ligang Cui, Kun Wang

**Affiliations:** 1 Institute of Medical Technology, Peking University Health Science Center, Beijing, 100191 China; 2 Department of Diagnostic Ultrasound, Peking University Third Hospital, Beijing, China; 3 CAS Key Laboratory of Molecular Imaging, Institute of Automation, Chinese Academy of Sciences, Beijing, China; 4 School of Artificial Intelligence, University of Chinese Academy of Sciences, Beijing, China; 5 Department of Ultrasound, The Second Hospital of Hebei Medical University, Shijiazhuang, China; 6 Department of Ultrasound, Beijing Friendship Hospital, Capital Medical University, Beijing, China; 7 Department of Ultrasound, Beijing Civil Aviation General Hospital, Beijing, China

**Keywords:** Superficial soft-tissue masses, Deep learning model, Ultrasound, Diagnosis, Computer-assisted diagnosis

## Abstract

**Background:**

Most of superficial soft-tissue masses are benign tumors, and very few are malignant tumors. However, persistent growth, of both benign and malignant tumors, can be painful and even life-threatening. It is necessary to improve the differential diagnosis performance for superficial soft-tissue masses by using deep learning models. This study aimed to propose a new ultrasonic deep learning model (DLM) system for the differential diagnosis of superficial soft-tissue masses.

**Methods:**

Between January 2015 and December 2022, data for 1615 patients with superficial soft-tissue masses were retrospectively collected. Two experienced radiologists (radiologists 1 and 2 with 8 and 30 years’ experience, respectively) analyzed the ultrasound images of each superficial soft-tissue mass and made a diagnosis of malignant mass or one of the five most common benign masses. After referring to the DLM results, they re-evaluated the diagnoses. The diagnostic performance and concerns of the radiologists were analyzed before and after referring to the results of the DLM results.

**Results:**

In the validation cohort, DLM-1 was trained to distinguish between benign and malignant masses, with an AUC of 0.992 (95% CI: 0.980, 1.0) and an ACC of 0.987 (95% CI: 0.968, 1.0). DLM-2 was trained to classify the five most common benign masses (lipomyoma, hemangioma, neurinoma, epidermal cyst, and calcifying epithelioma) with AUCs of 0.986, 0.993, 0.944, 0.973, and 0.903, respectively. In addition, under the condition of the DLM-assisted diagnosis, the radiologists greatly improved their accuracy of differential diagnosis between benign and malignant tumors.

**Conclusions:**

The proposed DLM system has high clinical application value in the differential diagnosis of superficial soft-tissue masses.

**Supplementary Information:**

The online version contains supplementary material available at 10.1186/s12916-023-03099-9.

## Background

Superficial soft-tissue masses refer to various benign and malignant masses occurring in the superficial skin layer, subcutaneous tissue layer (fat, fibrous connective tissue and blood vessels), and muscle tissue layer [1] and present as subcutaneous masses of different sizes during palpation, which may be accompanied by pain, swelling, and dysfunction [2]. The annual incidence of superficial soft-tissue masses is about 3‰, and the incidence has increased in recent years [3]. Most are benign tumors, and very few are malignant tumors (less than 1%) [4]. However, both benign and malignant persistent growth can cause pain and discomfort. Malignant masses that continue to develop may cause complications, such as pathological fractures, and may diffuse and become life-threatening. In clinical practice, compared with benign and malignant classification, the difficulty in the diagnosis of soft-tissue masses lies in the benign classification, because benign has more than 70 subtypes and rarely displays typical imaging signs of each subtype in individuals, and the accuracy of diagnosis is strongly influenced by the radiologist's experience, so the accuracy rate of the most radiologists is less than 70%. Therefore, early detection and correct diagnosis are of great significance for the reasonable treatment and prognosis of superficial soft-tissue masses.

CT, MRI, and ultrasound can all be used for the examination of superficial soft-tissue masses. Among them, CT [5] has an ideal localization function and can show the relationship between tumor size, location, boundary, and surrounding tissues. However, its resolution on soft-tissue is low, and sometimes it is difficult to be qualitative, and it is also radioactive. Although MRI [6] can clarify the soft tissue structure, it is expensive and requires a long scanning time [7], both of which are not conducive for the diagnosis of superficial soft-tissue masses in clinical practice. In contrast, ultrasound has good soft-tissue resolution, is non-invasive, safe, non-radioactive, inexpensive, can be repeated multiple times, and can have a clinical palpation function during the examination, which is an incomparable advantage over other imaging methods, so it is the best method for the initial diagnosis of superficial soft-tissue masses [8–13]. However, in clinical practice, the diagnosis of superficial soft-tissue masses mainly depends on the experience and ability of the radiologist, which is subjective. Therefore, an automated tool that can provide screening and auxiliary diagnosis of superficial soft-tissue masses is necessary to improve the diagnostic efficiency and accuracy of radiologists.

Different from traditional methods, deep learning radiomics (DLR) is an emerging technology based on data-driven learning, which can mine a large number of quantitative and high-throughput features that are difficult for human eyes to recognize from medical images for diagnosis and prognosis [14, 15]. However, the lesion edge in ultrasonic images is fuzzy, which is greatly affected by the operator, and it is difficult to manually define and extract features, and the reliability is poor [15]. DLR can automatically extract medical image features by using a deep neural network structure, so the most significant advantage of DLR is that it does not need to manually extract features [14, 15].

There have been many studies on deep learning radiomics based on ultrasonic images [15–21]. All these studies have obtained satisfactory results, indicating that the establishment of a deep learning model is conducive to more efficient ultrasonic diagnosis. However, as far as we know, there is only one study [22] that applies artificial intelligence (AI) to ultrasound images to distinguish and identify superficial soft-tissue masses. This study was conducted on 419 patients in a single center, but its model had a small amount of data, simple model results, and poor benign identification performance. Therefore, more comprehensive studies with larger data cohorts are warranted to explore the differentiating performance of ultrasound-based DLR for superficial soft-tissue masses.

In this study, we retrospectively collected 1615 cases of superficial soft-tissue masses and aim to propose a new ultrasound deep learning model system consisting of two deep learning models (DLM-1 and DLM-2) for the classification and diagnosis of superficial soft-tissue masses. DLM-1 is trained to distinguish between benign and malignant masses, and DLM-2 is trained to classify the five most common benign superficial soft-tissue masses: lipomyoma, hemangioma, neurinoma, epidermal cyst, and calcifying epithelioma. Furthermore, we found data on superficial soft-tissue masses from two hospitals as an external test cohort to validate the performance of the model. In addition, in order to further verify the clinical application value of the model, we compared the DLM with the radiologists.

## Methods

### Patients

In this study, we retrospectively collected data for a total of 1615 patients with superficial soft-tissue masses from Peking University Third Hospital and two other hospitals from January 2015 to December 2022. This study was approved by the Institutional Ethics Committee (approval number: S2022674), and the need to obtain informed consent from patients was waived.

All effective cases included in the study must have pathological biopsy (histopathological findings) results as a factual basis for the type of mass to be objective.

Inclusion criteria were as follows: (a) confirmed by histopathological findings of puncture biopsy or surgical excision; (b) the image is free from puncture needles and other external foreign bodies; (c) both two-dimensional grayscale images and color Doppler flow imaging (CDFI) images; (d) clear images with typical features.

Exclusion criteria were as follows: (a) no histopathological findings; (b) interference by puncture needles and other external bodies; (c) only one image with two-dimensional grayscale image and CDFI; (d) unqualified ultrasonic images; (e) soft-tissue benign masses in addition to the five benign masses studied in this study.

In this study, 20% of patients were randomly selected to be in the independent validation cohort, resulting in a 4:1 ratio of trained and validated patients. Stratified random sampling was used to ensure that model selections for the training and validation cohorts in this study were completely isolated, with a consistent proportion of patients responding and not responding.

### Acquisition and analysis of US findings

For each patient, we collected 5–8 frames of grayscale image and CDFI for screening suitable images and finally used one grayscale image and one CDFI for training and evaluation of the DLM. Most of the research was done using a 7–14 MHz linear array probe for image acquisition on a HITACHI AIRETT 70 or GE LOGIQ E9 system under the default parameter conditions of the instrument. At the same time, comparative scan and dynamic scan should be carried out when the image is collected to compare and dynamically observe the boundary and scope of the lump. If the lump is deep or large, appropriate pressure should be applied. We ensured that each case contained at least one grayscale image and one CDFI.

### Deep learning diagnostic and scoring models

**Fig. 1:**
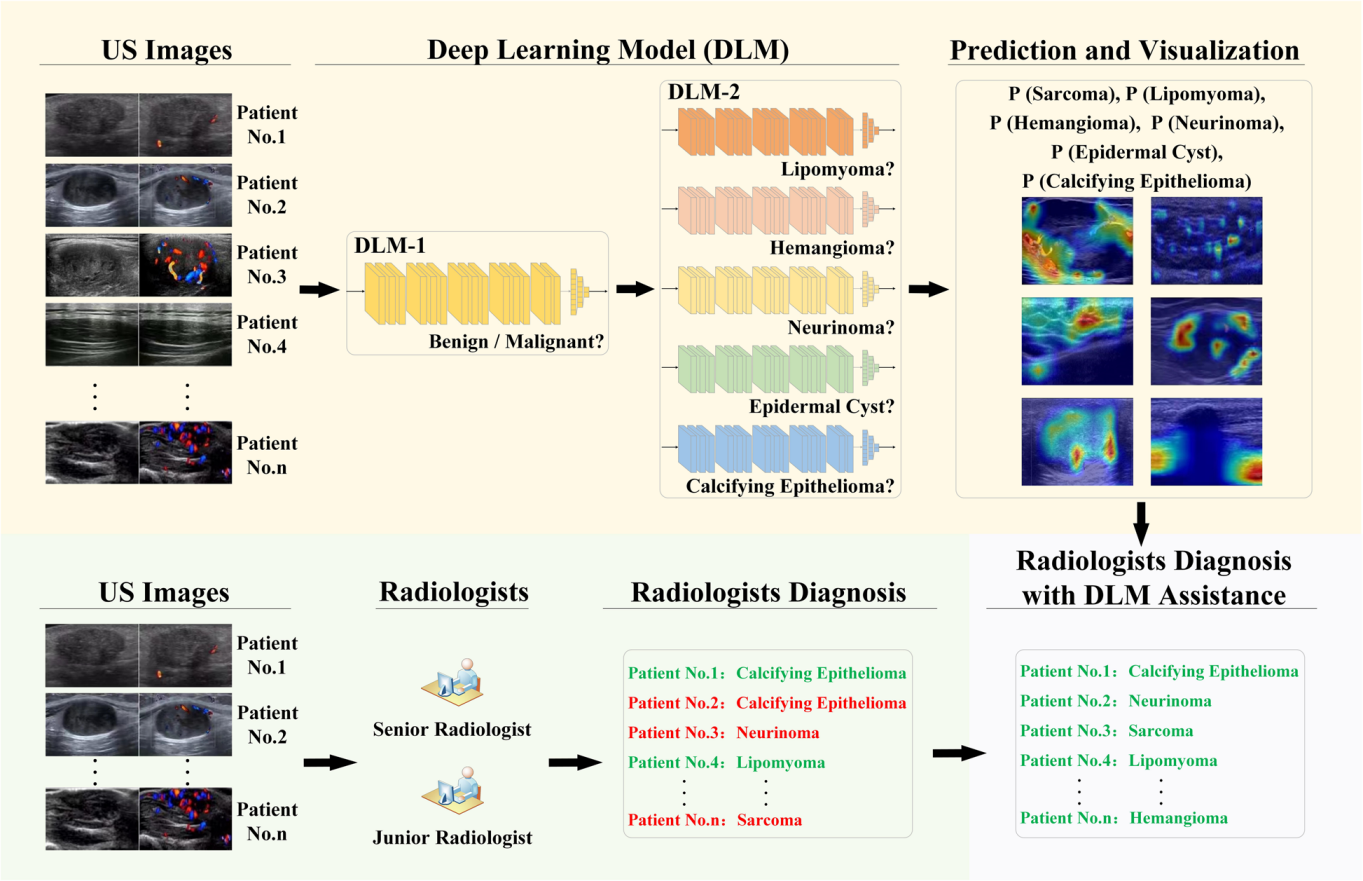
The structure of deep learning model system. For each test case, our model utilizes ultrasound images as inputs each time, outputs superficial soft-tissue masses diagnostic task-related predictive probabilities and corresponding heatmaps to compare with and assist radiologists

A deep learning model system based on ultrasound images, including two deep learning models (DLM-1 and DLM-2), was developed for the differential diagnosis of superficial soft-tissue masses (Fig. 1). DLM-1 was used to distinguish between benign and malignant masses. DLM-2 consisted of five sub-models (SM-1, SM-2, SM-3, SM-4, and SM-5 used to identify lipomyoma, hemangioma, neurinoma, epidermal cyst, and calcifying epithelioma under benign conditions, respectively). All of these models are similar in structure to ConvNeXt networks [23, 24] (see Additional file 1: Figure S1 and Method S1), with the only difference being the fully connected layer, to which we made some simple modifications to so that the network can adapt to current classification problems (see Additional file 1: Table S1). The input to each model is a grayscale ultrasound image or a CDFI image. The output of each model is the probability of each category from 0 to 1.

When applying these deep learning models, the region of interest (ROI) of each ultrasonic image is first extracted manually to avoid unnecessary text and graphic interference. Then, the ROI frame is adjusted to 470 × 280 based on the average size. In the training process, a series of data demonstration operations, including random scaling, random clipping, random flipping, and normalization, are needed to overcome overfitting. During the test, we directly resized each image to 470 × 280 and normalized each image in the same way as the normalization during the training. First, DLM-1 is applied to diagnose whether the sample is benign or malignant. If the sample is determined to be benign, further diagnosis is made by DLM-2, and the sub-model with the highest score gives the diagnosis.

The training cohort (*n* = 618) was used to train the model, the validation cohort (*n* = 154) was used to select the training hyperparameters and the best model during the training, and the test cohort A (*n* = 156) and the test cohort B (*n* = 123) were used to test the generalization performance of the model. It is important to note that in this study, the test queue was completely isolated from the training and model selection, so that they could be treated as two separate data cohorts. We used the pre-processed network weights on the ImageNet data cohort [25–27] as the initial weights. Finally, ultrasonic images were used to fine-tune the network weights. We used the same strategy to train SM-1, SM-2, SM-3, SM-4, SM-5, and DLM-1 (see Additional file 1: Method S2).

### Radiologist study

We compared the results of the DLM for the identification of benign and malignant masses and the classification of benign masses with the diagnoses of two radiologists of different seniority with 30 and 8 years of clinical experience (Radiological-1 and Radiological-2). In the reader study, each radiologist evaluated grayscale and Doppler images of 58 patients in an internal test cohort, regardless of clinical history or patient demographics, and recorded his image-only diagnosis. According to the comparison results, the performance and clinical application value of the DLM were obtained.

### Statistical analysis

Accuracy, specificity, sensitivity, positive predictive value (PPV), negative predictive value (NPV), and f1-score were calculated to show the diagnostic performance of the DLM (see Additional file 1: Method S3). The *χ*^2^ test for independence was used to calculate *P* values for categorical variables (gender and mass type), and the one-way ANOVA was used to calculate *P* values for quantitative variables (age). The area under the receiver operating characteristic (ROC) curve (AUC) was used to estimate the performance of the DLM. For all tests mentioned above, a *P* value of < 0.05 was considered significant. The statistical analyses were performed using Python and SciPy.

## Results

### Baseline characteristics

**Fig. 2:**
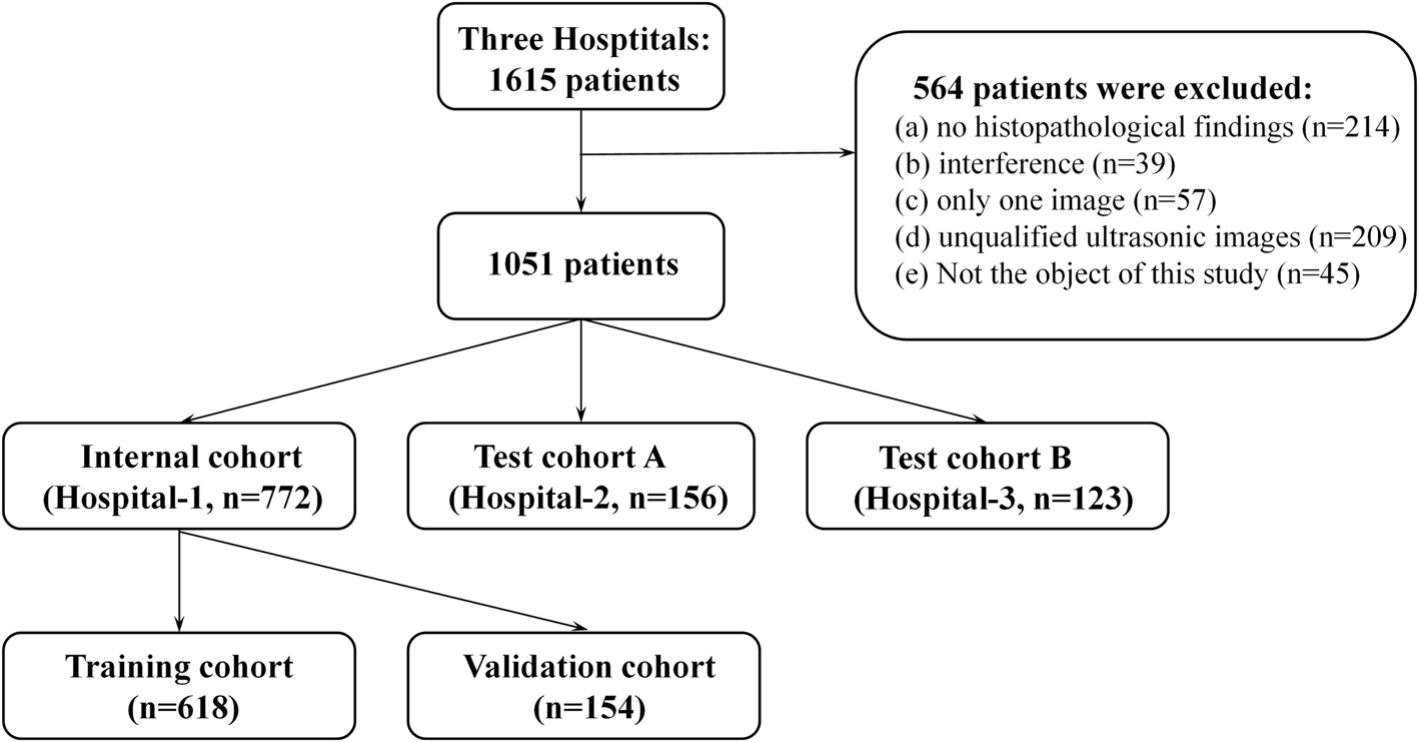
Patient selection flowchart

In this study, we retrospectively collected data for a total of 1615 patients with superficial soft-tissue masses, of which 564 patients were excluded due to exclusion criteria: (a) 214 cases, (b) 39 cases, (c) 57 cases, (d) 209 cases, (e) 45 cases. Finally, a total of 1051 cases were included for model training and verification (Fig. 2).

The data cohort of the Third Hospital of Beijing Medical University (Hospital-1 = 772) was randomly assigned as the training cohort (*n* = 618), the validation cohort (*n* = 154), the test cohort A (Hospital-2 = 156) of Beijing Civil Aviation General Hospital, and the test cohort B (Hospital-3 = 123) of Beijing Friendship Hospital Affiliated to Capital Medical University. Baseline characteristics of these patients are summarized in Table 1. There were certain statistical differences among benign data cohorts, which may be caused by large sample data and different hospitals.

**Table 1:** Patient and tumor baseline characteristics

		Internal cohort		Test cohort A	Test cohort B	*P*
		Training cohort	Validation cohort			
Subject		618	154	156	123	
Malignant						
Age		49.1 (± 16.1)	51.0 (± 12.2)	47.4 (± 12.0)	49.1 (± 16.1)	0.91
Gender	Male	17 (53.1%)	4 (50.0%)	2 (40.0%)	4 (40.0%)	0.431
	Total	32	8	5	10	
Benign						
Age		33.9 (± 9.0)	32.7 (± 7.9)	32 (± 7.5)	33.9 (± 9.0)	0.02
Gender	Male	288 (49.2%)	69 (47.3%)	80 (53.0%)	59 (52.2%)	< 0.001
Type						0.028
	Lipomyoma	121 (20.6%)	30 (20.5%)	40 (26.5%)	28 (24.8%)	
	Hemangioma	120 (20.5%)	30 (20.5%)	16 (10.5%)	20 (17.7%)	
	Neurinoma	120 (20.5%)	30 (20.5%)	25 (16.6%)	18 (15.9%)	
	Epidermal cyst	160 (27.3%)	40 (27.4%)	45 (29.8%)	27 (23.9%)	
	Calcifying epithelioma	65 (11.1%)	16 (11.1%)	25 (16.6%)	20 (17.7%)	
	Total	586	146	151	113	

Data are presented as *n* (%) or mean ± SD

### Performance of DLM-1

DLM-1 is used to distinguish between benign and malignant soft-tissue masses. In the training cohort, DLM-1 had an AUC of 0.915 (95% CI: 0.871, 0.950) and an ACC of 0.875 (95% CI: 0.854, 0.896). In the validation cohort, the AUC of DLM-1 reached a staggering 0.992 (95% CI: 0.980, 1.0), and the ACC was 0.987 (95% CI: 0.968, 1.0).

**Fig. 3:**
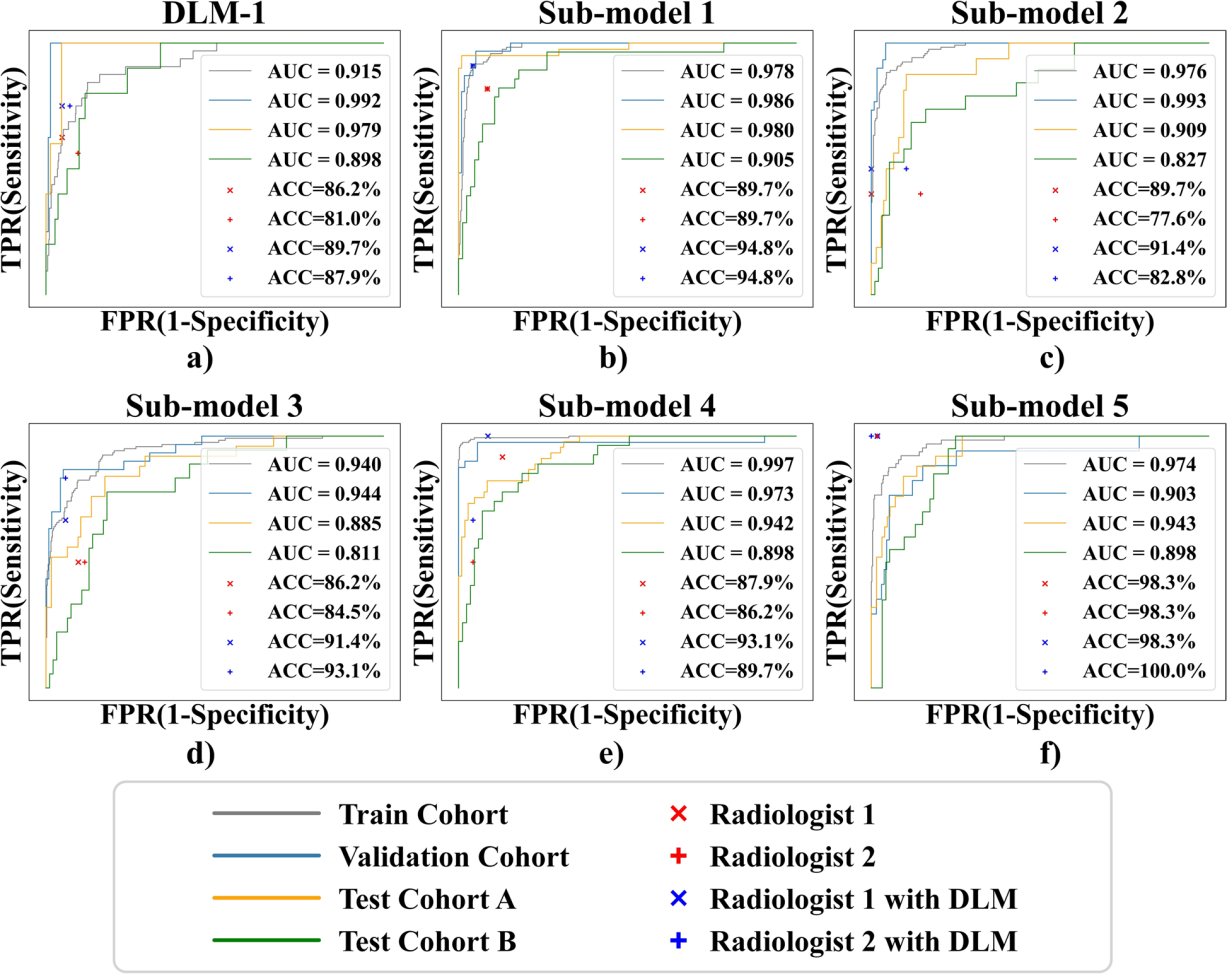
ROC curves of the DLM-1 and the DLM-2. ROC curves of the DLM in the train cohort, validation cohort, test cohort A, and test cohort B. **a** ROC curves of DLM-1. **b** ROC curves of lipomyoma in DLM-2. **c** ROC curves of hemangioma in DLM-2. **d** ROC curves of neurinoma in DLM-2. **e** ROC curves of epidermal cyst in DLM-2. **f** ROC curves of calcifying epithelioma in DLM-2. ROC, receiver operating characteristic curve; AUC, area under the receiver operator characteristic curve; DLM, deep learning model

DLM-1 also performed well in two external test cohorts, with AUCs of 0.979 (95% CI: 0.952, 1.0) and 0.898 (95% CI: 0.827, 0.959), respectively (see Table 2 for all quantitative indicators of DLM-1, and the ROC curves are shown in Fig. 3a).

**Table 2:** Diagnostic performance of DLM-1

	Training cohort	Validation cohort	Test cohort A	Test cohort B
AUC	0.915 [0.871, 0.95]	0.992 [0.98, 1.0]	0.979 [0.952, 1.0]	0.898 [0.827, 0.959]
ACC (%)	87.5 [84.7, 90.0]	98.7 [95.4, 99.8]	97.4 [93.6, 99.3]	91.0 [84.4, 95.4]
Sensitivity (%)	84.4 [67.2, 94.7]	100.0 [63.1, 100.0]	40.0 [5.3, 85.3]	20.0 [2.5, 55.6]
Specificity (%)	87.7 [84.8, 90.2]	98.6 [95.2, 99.8]	99.3 [96.4, 100.0]	97.3 [92.4, 99.4]
PPV (%)	27.3 [22.4, 32.8]	80.0 [50.2, 94.1]	66.7 [17.7, 94.9]	40.0 [11.2, 78.0]
NPV (%)	99.0 [97.9, 99.6]	100.0 [100.0, 100.0]	98.0 [96.1, 99.0]	93.2 [91.0, 94.9]
F1-score	0.412 [0.318, 0.5]	0.889 [0.706, 1.0]	0.5 [0, 0.857]	0.267 [0, 0.5]

Data in brackets are the 95% confidence interval

*Abbreviations*: *AUC* area under the receiver operating characteristic curve, *ACC* accuracy, *PPV* positive predict value, *NPV* negative predict value, *DLM* deep learning model, training cohort (*n* = 617 individuals), validation cohort (*n* = 155 individuals), test A cohort (*n* = 156 individuals), test B cohort (*n* = 122 individuals)

### Performance of DLM-2

DLM-2 is used to distinguish the five most common benign superficial soft-tissue masses, including lipomyoma, hemangioma, neurinoma, epidermal cyst, and calcifying epithelioma. For the model training cohort, validation cohort, and test cohorts A and B, the AUCs of lipomyoma classification were 0.980, 0.986, 0.980, and 0.905, respectively. The AUCs of hemangioma classification were 0.976, 0.993, 0.909, and 0.827, respectively. The AUCs of neurinoma were 0.940, 0.944, 0.885, and 0.811, respectively. The AUCs of epidermal cyst classification were 0.997, 0.973, 0.942, and 0.898, respectively. The AUCs of calcifying epithelioma were 0.974, 0.903, 0.943, and 0.898, respectively. The ROC curves are shown in Fig. 3b–f. Table 3 summarizes the various performance indicators of the 95% CI of DLM-2.

**Table 3:** Diagnostic performance of DLM-2

	AUC	ACC (%)	Sensitivity (%)	Specificity (%)	PPV (%)	NPV (%)	F1-score
Lipomyoma							
Training cohort	0.98 [0.954, 1.0]	94.3 [92.6, 95.9]	90.8 [86.1, 95.1]	95.3 [93.4, 96.8]	83.2 [77.3, 88.5]	97.6 [96.3, 98.7]	0.869 [0.825, 0.904]
Validation cohort	0.986 [0.973, 0.997]	95.3 [92.6, 98.0]	83.9 [71.9, 93.8]	98.3 [96.4, 100.0]	92.9 [84.2, 100.0]	95.8 [92.7, 98.4]	0.881 [0.8, 0.947]
Test cohort A	0.98 [0.954, 1.0]	84.1 [78.8, 88.7]	95.0 [88.9, 100.0]	80.2 [73.7, 86.0]	63.3 [53.1, 73.8]	97.8 [95.2, 100.0]	0.76 [0.674, 0.832]
Test cohort B	0.905 [0.847, 0.956]	78.6 [72.3, 84.8]	96.4 [89.5, 100.0]	72.6 [64.9, 80.2]	54.0 [42.6, 65.9]	98.4 [95.2, 100.0]	0.692 [0.59, 0.786]
Hemangioma							
Training cohort	0.976 [0.966, 0.985]	90.6 [88.5, 92.6]	92.5 [88.2, 96.3]	90.1 [87.8, 92.2]	70.7 [64.5, 76.7]	97.9 [96.7, 99.0]	0.801 [0.756, 0.844]
Validation cohort	0.993 [0.986, 0.999]	93.9 [90.5, 96.6]	70.0 [55.9, 82.9]	100.0 [100.0, 100.0]	100.0 [100.0, 100.0]	92.9 [89.1, 96.2]	0.824 [0.717, 0.906]
Test cohort A	0.909 [0.851, 0.957]	79.5 [74.2, 84.8]	87.5 [71.4, 100.0]	78.5 [72.7, 84.6]	32.6 [21.2, 45.2]	98.1 [95.6, 100.0]	0.475 [0.333, 0.606]
Test cohort B	0.827 [0.735, 0.909]	72.3 [65.2, 80.4]	73.7 [57.1, 90.0]	72.0 [64.3, 80.4]	35.0 [22.9, 48.4]	93.1 [87.7, 97.5]	0.475 [0.333, 0.604]
Neurinoma							
Training cohort	0.94 [0.92, 0.96]	85.1 [82.7, 87.7]	92.5 [88.3, 96.3]	83.2 [80.3, 86.2]	58.7 [53.1, 64.9]	97.7 [96.4, 99.0]	0.718 [0.669, 0.766]
Validation cohort	0.944 [0.902, 0.978]	89.1 [85.0, 93.2]	86.7 [76.0, 96.4]	89.7 [85.0, 94.0]	68.4 [55.2, 80.0]	96.3 [93.4, 99.1]	0.765 [0.656, 0.847]
Test cohort A	0.885 [0.816, 0.94]	82.8 [77.5, 87.4]	84.0 [70.8, 95.8]	82.5 [76.4, 88.2]	48.8 [35.7, 61.0]	96.3 [93.1, 99.1]	0.618 [0.491, 0.716]
Test cohort B	0.811 [0.716, 0.895]	65.2 [58.0, 72.3]	77.8 [60.0, 94.1]	62.8 [54.2, 70.3]	28.6 [18.0, 38.5]	93.7 [88.1, 98.4]	0.418 [0.286, 0.527]
Epidermal cyst							
Training cohort	0.997 [0.993, 1.0]	98.5 [97.6, 99.1]	97.5 [95.2, 99.4]	98.8 [97.9, 99.5]	96.9 [94.4, 98.8]	99.1 [98.3, 99.8]	0.972 [0.956, 0.986]
Validation cohort	0.973 [0.928, 0.998]	95.2 [92.5, 98.0]	87.5 [78.0, 95.1]	98.1 [95.8, 100.0]	94.6 [88.1, 100.0]	95.5 [91.7, 98.2]	0.909 [0.842, 0.958]
Test cohort A	0.942 [0.911, 0.969]	84.8 [79.5, 89.4]	51.1 [38.3, 63.8]	99.1 [97.2, 100.0]	95.8 [88.5, 100.0]	82.7 [76.8, 87.7]	0.667 [0.54, 0.767]
Test cohort B	0.898 [0.839, 0.942]	82.1 [75.9, 87.5]	33.3 [17.9, 48.1]	97.6 [94.7, 100.0]	81.8 [58.3, 100.0]	82.2 [75.8, 88.1]	0.474 [0.286, 0.622]
Calcifying epithelioma							
Training cohort	0.974 [0.958, 0.986]	91.3 [89.2, 93.2]	90.6 [84.2, 96.0]	91.3 [89.2, 93.5]	56.3 [48.3, 64.2]	98.8 [97.9, 99.6]	0.695 [0.625, 0.759]
Validation cohort	0.903 [0.816, 0.967]	85.8 [81.8, 90.5]	82.4 [66.7, 95.7]	86.3 [81.5, 91.0]	43.8 [29.3, 57.7]	97.4 [94.8, 99.2]	0.571 [0.417, 0.692]
Test cohort A	0.943 [0.909, 0.973]	90.1 [86.1, 94.0]	60.0 [42.9, 76.9]	96.0 [93.1, 98.4]	75.0 [58.8, 89.5]	92.4 [88.7, 96.1]	0.667 [0.514, 0.787]
Test cohort B	0.898 [0.848, 0.942]	87.5 [82.1, 92.0]	50.0 [32.0, 69.6]	95.7 [91.7, 98.9]	71.4 [50.0, 91.7]	89.8 [84.5, 94.2]	0.588 [0.4, 0.744]

Data in brackets are the 95% confidence interval

*Abbreviations*: *AUC* area under the receiver operating characteristic curve, *ACC* accuracy, *PPV* positive predict value, *NPV* negative predict value, *DLM* deep learning model, training cohort (*n* = 584 individuals), validation cohort (*n* = 148 individuals), test cohort A (*n* = 151 individuals), test cohort B (*n* = 112 individuals)

### Results of radiologist study

In this study, two radiologists of different seniority (Radiologist-1: 30 years and Radiologist-2: 8 years of clinical experience) identified 58 benign and malignant tumors in the validation cohort (including 16 malignant sarcomas, 10 lipomyoma, 12 hemangiomas, 6 neurinomas, 10 epidermal cysts, and 4 calcifying epithelioma), and the results were 86.2% (50/58) and 81% (47/58), respectively. They classified the benign mass correctly 71.4% (30/42) and 59.5% (25/42), respectively.

Without knowing the exact results of the cases, the DLM assisted the two radiologists to re-diagnose the previous 58 cases: the differential results of benign and malignant masses were 89.7% (52/58) and 87.9% (51/58), respectively. The results of benign mass classification were 80.9% (34/42) and 73.8% (31/42), respectively. ROC curves of DLM validation cohort compared with the two radiologists (Fig. 3b–f).

### Interpretability of the DLM

**Fig. 4:**
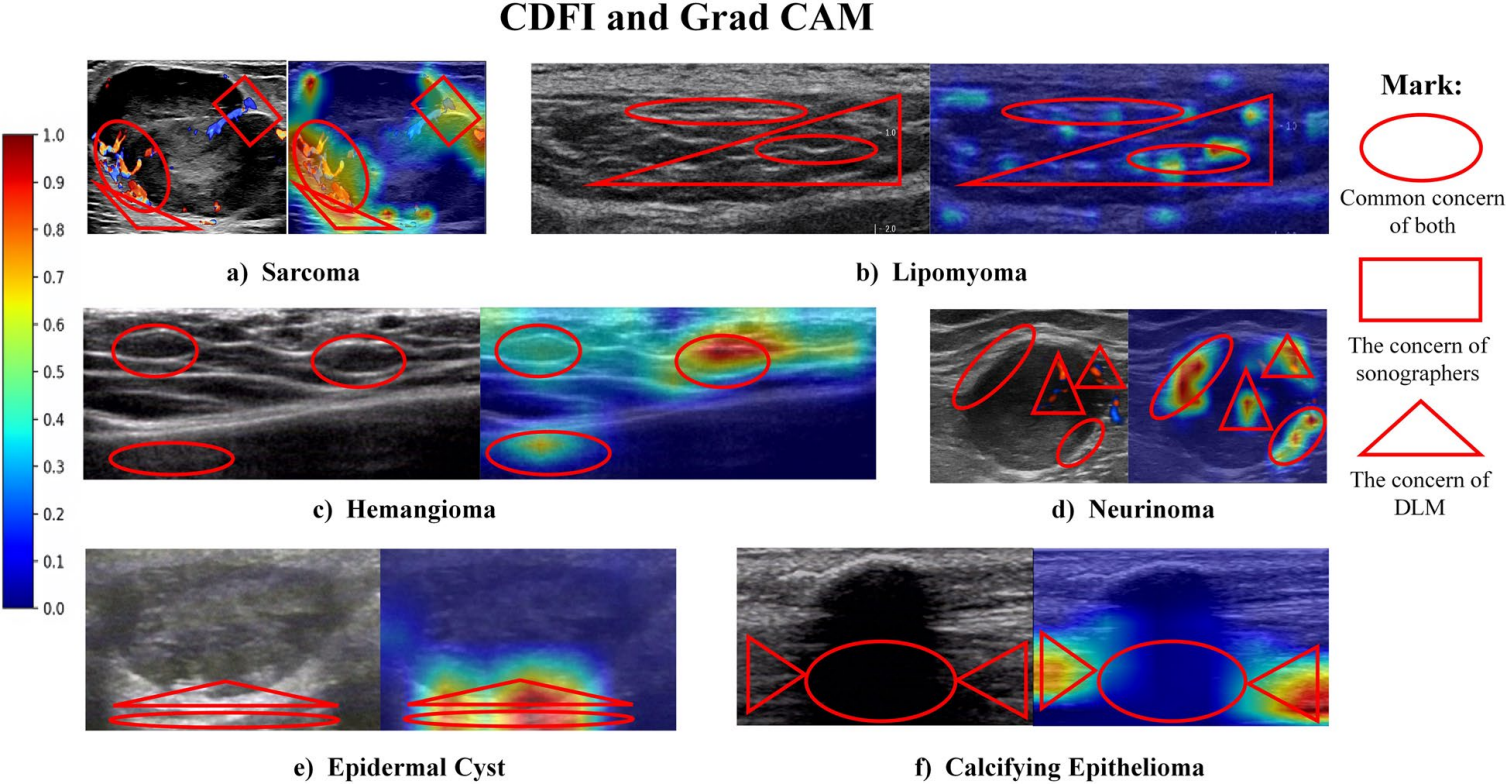
Visualization of the DLM using the Grad-CAM. CDFI and activation maps of 6 types of lumps are shown. The strong response areas (red areas) are also the areas the DLM paid more attention to, which also means that these areas are more valuable for response prediction. The ovals represent the common areas of concern of the radiologist and the DLM; the squares represent the areas of greater concern of the radiologist; the triangles represent the areas of greater concern of the DLM. CDFI, color Doppler flow imaging; DLM, deep learning model

In order to explore the interpretability of the DLM, we used gradient-weighted class activation mapping (Grad CAM) to visualize it [28] and found the areas of most concern of the DLM through a visualization algorithm, as shown in Fig. 4.

We randomly selected 130 patients in the internal dataset, used Grad CAM to display the areas of most concern of the DLM system, and then compared it with the areas of most concern of the two radiologists of different seniority. We found the following: in Radiologist-1, 28.4% (37/130) of the two areas of concern coincided completely; most overlapped 56.2% (73/130); a few overlapped 13.1% (17/130); there was 2.3% (3/130) that did not coincide at all. In Radiologist-2, there was 23.1% (30/130) that did not coincide at all. 52.3% (68/130) mostly overlapped; 17.7% (23/130) overlapped in a small part; 6.9% (9/130) did not overlap at all. Table 4 summarizes the areas of concern of level of coincidence between the DLM and the two radiologists.

**Table 4:** The areas of concern of level of coincidence between DLM and radiologists

Level of coincidence	Complete (100%)	Most (50–99%)	Partial (1–49%)	Not at all (0)
Radiologist-1				
Lipomyoma	36.4% (8/22)	54.5% (12/22)	9.1% (2/22)	0 (0/22)
Hemangioma	25.0% (5/20)	60.0% (12/20)	10.0% (2/20)	5.0% (1/20)
Neurinoma	13.8% (4/29)	72.4% (21/29)	20.7% (6/29)	0 (0/29)
Epidermal cyst	20.7% (6/29)	58.6% (17/29)	10.3% (3/29)	3.5% (1/29)
Calcifying Epithelioma	55.0% (11/20)	30.0% (6/20)	15.0% (3/20)	0 (0/20)
Sarcoma	30.0% (3/10)	50.0% (5/10)	10.0% (1/10)	10.0% (1/10)
Total	28.4% (37/130)	56.2% (73/130)	13.1% (17/130)	2.3% (3/130)
Radiologist-2				
Lipomyoma	27.3% (6/22)	68.2% (15/22)	4.6% (1/22)	0 (0/22)
Hemangioma	25.0% (5/20)	35.0% (7/20)	25.0% (5/20)	15.0% (3/20)
Neurinoma	6.9% (2/29)	72.4% (21/29)	27.6% (8/29)	3.5% (1/29)
Epidermal cyst	13.8% (4/29)	55.2% (16/29)	13.8% (4/29)	6.9% (2/29)
Calcifying epithelioma	50.0% (10/20)	30.0% (6/20)	15.0% (3/20)	5.0% (1/20)
Sarcoma	30.0% (3/10)	30.0% (3/10)	20.0% (2/10)	20.0% (2/10)
Total	23.1% (30/130)	52.3% (68/130)	17.7% (23/130)	6.9% (9/130)

## Discussion

This study evaluated the performance of a DLM system in the differential diagnosis of superficial soft-tissue masses, especially its value for less experienced and experienced radiologists. The DLM-assisted diagnosis was significantly helpful for the two radiologists.

DLM-1 and DLM-2 are two deep learning diagnostic models. DLM-1 was trained to distinguish between benign and malignant masses, and it can be seen from Table 2 that DLM-1 showed excellent performance. In the validation cohort, the AUC of DLM-1 reached an astonishing 0.992 (95% CI: 0.980, 1.0), and the ACC was 0.987 (95% CI: 0.968, 1.0), which highly indicated that the model was more accurate than the clinician in distinguishing benign from malignant masses. DLM-2 was trained to classify the five most common benign masses (lipomyoma, hemangioma, neurinoma, epidermal cyst, calcifying epithelioma), and the AUCs in the validation cohort were 0.986, 0.993, 0.944, 0.973, and 0.903, respectively. In test cohort B, the DLM performed slightly worse because the ultrasonic images were taken on machines of different make and model from those used in the other two centers. As can be seen from the above data, all the performance indexes of DLM-2 were about 0.9, indicating that DLM-2 had a strong ability in classifying five kinds of benign soft-tissue masses. The combination of the two models can accurately diagnose soft-tissue masses. It can be seen that deep learning is not subjective like humans, so it can accurately and stably carry out reasonable classification, avoiding the problem of missed diagnosis and misdiagnosis caused by the subjective judgment of disease types.

In the radiologist study, under the condition of DLM-assisted diagnosis, the accuracy of diagnosis by the radiologist was greatly improved in both benign and malignant differentiation and benign classification, especially in benign classification. However, only in the diagnosis of calcifying epithelioma, the effect of elevation is not good; because the clinical radiologist’s diagnosis accuracy is already high, DLM-assisted with no significant improvement. Also, with the help of the DLM, junior radiologists can achieve the diagnostic accuracy of senior radiologists. Thus, the DLM has certain clinical application value in assisting radiologists in the diagnosis of soft-tissue masses.

We used Grad CAM to visualize the DLM. When comparing the areas of most concern identified by the DLM and those identified by the radiologists, we found there were many common areas of concern (the reasons why the proportion of complete or most overlap between the two was more than 75%). For example, (1) for malignant masses [29], both of them were very concerned about the rich blood flow inside the lesion (Fig. 4a); (2) for lipomyoma [30], both of them focused on the strong echo lines inside the lesion (Fig. 4b); (3) for hemangioma , both of them paid much attention to the obvious internal honeycomb structure and the enhanced echo behind the lesion (Fig. 4c); (4) for neurinoma [31, 32], both of them focused on the “bright cap sign” of the lesions (Fig. 4d); (5) for epidermal cyst [33, 34], both of them were very concerned about the enhancement of the echo behind the lesion (Fig. 4e); and (6) for calcifying epithelioma [35, 36], both of them focused on the obvious attenuation of the echo behind the lesion (Fig. 4f).

In addition, the two had many different concerns. For example, (1) for malignant masses, the radiologists focused on sharp but irregular edges of the lesion, while the DLM focused on the hyperechoic wrapping of unequal thickness around the lesion, which represents a large number of small interfaces after infiltration, which the radiologists did not pay sufficient attention to (Fig. 4a); (2) for lipomyoma, when there were not many thick lines, the DLM paid more attention to the thick lines; when there were many thin lines, the DLM paid more attention to the two thin lines that were very close together. More lines and fine lines indicate that there are many normal fascia lines in the lesion, meaning it is more likely to be benign, and there are fewer fascia lines in the malignant mass, which is really not generally paid attention to by ultrasound doctors (Fig. 4b). (3) For neurinoma, the DLM paid more attention to blood flow signals inside the lesions, indicating solid nodules (Fig. 4d); (4) for epidermal cyst, the DLM’s focus was on the beginning of the lateral sound shadow, which means that the site is smooth and not easy for the radiologist to see at a glance (Fig. 4e).

We found that there were many similarities and differences between the DLM area of concern and the signs of the radiologist. For the similarities, the rationality and feasibility of the model can be further confirmed. At the same time, it can also help doctors quickly find the focus of the lesion area. For different points, it can provide clinicians with lesion areas to focus on in other points and provide new ideas for clinical diagnosis. This phenomenon may come from this reason: in terms of image labeling, we did not cover and sketch the boundary details of the lesion as traditional labeling did, but chose to use a wide range of field of view to intercept, which gave the model more space for self-discovery and learning. Compared with the traditional model, which only saw the details that the doctor wanted the model to see, our method may enable the model to discover the details that the doctor did not find.

Currently, the only relevant work is an artificial intelligence model proposed by Benjamin Wang et al. [22] to distinguish soft-tissue masses. Their model does a good job of distinguishing benign from malignant. However, their work has many limitations. First, the number of cases they collected was small (*n* = 419), and there were many cases without two-dimensional and color Doppler ultrasound images. Second, they had no external test cohort and were not verified by other hospital data, so the model performance results were not convincing. Third, although their model did a good job of distinguishing benign from malignant, it failed completely to identify the three benign masses and did not even mention benign differentiation in the study’s conclusion. Finally, the artificial intelligence model applied in this study is simple in structure and low in efficiency, with low value for clinical application. However, we propose and verify that a DLM that addresses these deficiencies well and achieves excellent performance, establishing a more effective and clinically applicable model for the differentiation of soft-tissue masses.

The main limitation of the study relates to the reader study design of two specialist radiologists. In the reader study, the radiologist could only interpret selected static two-dimensional grayscale and CDFI images. In practice, radiologists can combine patient history, clinical symptoms, and real-time dynamic image information to obtain diagnosis results. The reader design of the study did not take this into account, which may have underestimated the performance of the radiologists. Another limitation is that due to the small number and wide variety of malignant cases, it is not possible to further distinguish malignant cases. In the future, we will use more clinical data collected to classify malignant masses, which may further improve the diagnostic performance of the DLM system.

## Conclusions

In summary, we propose a new ultrasound deep learning model system, including two deep learning models (DLM-1 and DLM-2), with good performance for the classification and diagnosis of superficial soft-tissue masses. If this model is applied clinically, it may help to improve the accuracy of classification and identification of soft-tissue mass by the radiologist. Furthermore, it is helpful for improving the diagnostic efficiency of soft-tissue masses in the physical examination screening scenario and has high clinical application value.

### Supplementary Information

**Additional file 1:** **Method S1.** [Basic principles and components of deep learning and neural networks]. **Method S2.** [Details of training strategy]. **Method S3.** [Statistic metric of deep learning models]. **Fig. S1.** [Detailed network architecture of deep learning models]. **Table S1.** [Detailed hyperparameter configuration of the deep learning models].

## Data Availability

Clinical and ultrasound images are not publicly available to protect patient privacy.

## Abbreviations

CDFI: Color Doppler flow imaging
DLM: Deep learning model
US: Ultrasound
ROC: Receiver operating characteristic curve
AUC: Areas under the curve
ROI: Regions of interest
SM: Sub-model
