# Supplementary material for: Deep learning models of ultrasonography significantly improved the differential diagnosis performance for superficial soft-tissue masses: a retrospective multicenter study
Source: BMC Med. 2023 Oct 26;21:405. doi: 10.1186/s12916-023-03099-9 (PMC10601110; doi:10.1186/s12916-023-03099-9)
Supplement: Supplementary file 1 — Additional file 1: Method S1. [Basic principles and components of deep learning and neural networks]. Method S2. [Details of training strategy]. Method S3. [Statistic metric of deep learning models]. Fig. S1. [Detailed network architecture of deep learning models]. Table S1. [Detailed hyperparameter configuration of the deep learning models]. [file 12916_2023_3099_MOESM1_ESM.docx]

**Additional file 1**

**Supplementary Material**

**Table of Contents**

**Methods**

Method S1. Basic principles and components of deep learning and neural networks

Method S2. Details of training strategy

Method S3. Statistic metric of deep learning models

**Figures**

Fig. S1. Detailed network architecture of deep learning models

**Tables**

Table S1. Detailed hyperparameter configuration of the deep learning models

**Method S1. Basic principles and components of deep learning and neural networks**

Deep learning is based on a deep neural network architecture to learn network weights for specific tasks. The common components are explained as follows.

**Convolutional layer.** The convolutional layer adopts some filters(kernels) with a sliding stride and a kernel size to calculate the weighted sum of the intensities of each point and its surrounding points of the input matrix. Its main function is to extract features from the input matrix. For example, assuming a input matrix $I=\left( \begin{matrix} I_{11} & I_{12} & I_{13} \\ I_{21} & I_{22} & I_{23} \\ I_{31} & I_{32} & I_{33} \end{matrix} \right)$ and a filter $K=\left( \begin{matrix} k_{11} & k_{12} \\ k_{21} & k_{22} \end{matrix} \right)$ with a kernel size of 2x2 and a stride of 1, the output of the convolutional layer is

$$F=conv(I, K)=\left( \begin{matrix} {I_{11}*k}_{11}+{I_{12}*k}_{12}{{+I}_{21}*k}_{21}+{I_{22}*k}_{22} & {I_{12}*k}_{11}+{I_{13}*k}_{12}{{+I}_{22}*k}_{21}+{I_{23}*k}_{22} \\ {I_{21}*k}_{11}+{I_{22}*k}_{12}{{+I}_{31}*k}_{21}+{I_{32}*k}_{22} & {I_{22}*k}_{11}+{I_{23}*k}_{12}{{+I}_{32}*k}_{21}+{I_{33}*k}_{22} \end{matrix} \right)$$

The output F is a channel of the feature map. By using multiple filters, multi-channel feature can be generated, which significantly improves the fitting ability of the convolutional layer. Through the continuous stacking of convolutional layers, the neural network can extract more abstract features.

**Depth-wise convolution layer.** Depth-wise Convolution (DWC) is that each convolution kernel only performs convolution on one channel of the input feature map. DWC operation is carried out in two-dimensional plane, so the number of channels does not change. For example, to perform DWC on a 5×5 pixel, three-channel input image (5×5×3), the number of convolution kernels is the same as the number of channels of the input image (one-to-one correspondence between channels and convolution kernels). Therefore, the three-channel image is processed to generate 3 feature maps (if there is same padding, the output size is the same as the input size, which is 5×5).

**Max pooling layer.** The maximum pooling layer is mainly used to perform feature selection and dimensionality reduction on the input feature map. Effective feature selection and dimensionality reduction can improve the robustness and reduce the complexity of the deep learning model. Assuming the feature map is $F=\left( \begin{aligned} \begin{matrix} 1 & 5 & \begin{matrix} 2 & 8 \end{matrix} \end{matrix} \\ \begin{matrix} 3 & 9 & \begin{matrix} 7 & 8 \end{matrix} \end{matrix} \\ \begin{matrix} 1 & 0 & \begin{matrix} 2 & 6 \end{matrix} \end{matrix} \\ \begin{matrix} 8 & 5 & \begin{matrix} 3 & 2 \end{matrix} \end{matrix} \end{aligned} \right)$, whose size is 4×4, and pooling sliding window is 2×2 with stride of 2. The pooling operation will divide the matrix *F* into four disjoint sub-matrixes of size 2×2, and the maximum value of each small matrix will be extracted to form the result matrix $P=\left( \begin{matrix} 9 & 8 \\ 8 & 6 \end{matrix} \right)$.

**Layer normalization layer.** The idea of layer normalization(LN) layer is to normalizes the input by calculating the mean and variance of all neurons in a certain layer. The following formula is the calculation method of the mean and variance in the LN algorithm.

$$u^{l}=\frac{1}{H}\sum_{i=1}^{H} a_{i}^{l},\sigma^{l}=\sqrt{\frac{1}{H}\sum_{i=1}^{H} {(a_{i}^{l}-u^{l})}^{2}}$$

Gains and biases are also added to each neuron to achieve a linear transformation, which is used before the activation function after normalization.

**Fully connected (FC) layer.** The fully connected layer can be described as a matrix multiplication plus one bias term, which is $FC\left( x \right)=Wx+b$. The fully connected layer is usually used at the top of the network, mainly to provide a global receptive field for the network, so as to overcome the shortcomings of the convolutional layer that has only limited receptive field. Through the fully connected layer, the features map can be fused and compressed simultaneously, and converted into the probability output of each category. In this study, the connected layers were used for two-category classification tasks.

**Activation function layer.** The operations in convolutional and fully connected layers can also be expressed in the form of matrix multiplication. Matrix multiplication is a linear operation, and it is often difficult to fit complex problems using only linear operations. To strengthen the nonlinear fitting ability of the neural network, the activation function layer needs to be used. In this study, we adopted GELU function layer

$$GELU(x)=0.5x(1+tanh(\sqrt{2/x}(x+0.044715x^{3})))$$

after most convolutional layers and fully connected layers in our networks.

**Global average pooling (GAP) layer.** The function of the GAP layer is to calculate the average intensity of all elements in each channel of a feature map, and finally output a feature vector which length is same as the number of the channels. The use of GAP layer can significantly reduce the number of weights in the network, and effectively alleviate overfitting. In this study, we used the GAP layer between the last convolutional layer and the connected layer.

**Method S2. Details of training strategy**

In our study, the same training strategy was used to train DLM-1 and DLM-2. The cross entropy was used as the loss function for optimization, which is

$$L\left( w \right)=\frac{1}{N}\sum_{n=1}^{N} \left[ y_{n}\log p_{n}+\left( 1-y_{n} \right)\log\left( 1-y_{n} \right) \right]$$

where *w* is the parameter of the model that needed to be trained; *N* is the number of training samples; $y_{n}$ represent the ground truth label; $p_{n}$ is the predicted probability. AdamW was used to update the model weights with batch size 8. The initial learning rate was set to 0.0005 and the weight decay was set to 0.0002. We set the maximum number of training epoch to 50.

During the training process, we continuously monitor the AUC or accuracy of the validation set. Finally, the model weights with the best performance in the validation set is selected as the final weights. We used the network weights pretrained on the Imagenet dataset as the initial weights. In particular, the last 1024 nodes FC layer was replaced with Kaiming uniform initialized weights. Our implementation of the deep learning model used the Pytorch 1.7 and Python 3.7.

**Method S3. Statistic metric of deep learning models**

In our study, the following 6 quantitative indicators including receiver operating characteristic curve (AUC), accuracy, sensitivity, specificity, positive predictive value (PPV) and negative predictive value (NPV) were used to evaluate the performance of our models. The formulas used to calculate these indicators are as follows:

1. AUC

$$AUC=\frac{\sum_{{ins}_{i}\in positiveclass} {rank}_{{ins}_{i}}-\frac{M\times(M+1)}{2}}{M\times N}$$

Where M, N are the number of positive samples and negative samples. ${rank}_{{ins}_{i}}$ is the serial number of sample i.

1. Accuracy

$$accuracy = \frac{TP+TN}{TP+TN+FP+FN}$$

2. Sensitivity

$$sensitivity = \frac{TP}{TP+FN}$$

3. Specificity

$$specificity = \frac{TN}{TN+FP}$$

4. PPV

$$PPV = \frac{TP}{TP+FP}$$

5. NPV

$$NPV = \frac{TN}{TN+FN}$$

1. F1-score

$$F1 score = \frac{2\cdot Precision\cdot Recall}{Precision+Recall}$$

$$Precision = \frac{True Positives}{True Positives+False Positives}$$

$$Recall = \frac{True Positives}{True Positives+False Negatives}$$

Where TP, TN, FP, FN are the number of true positive samples, true negative samples, false positive samples and false negative samples.


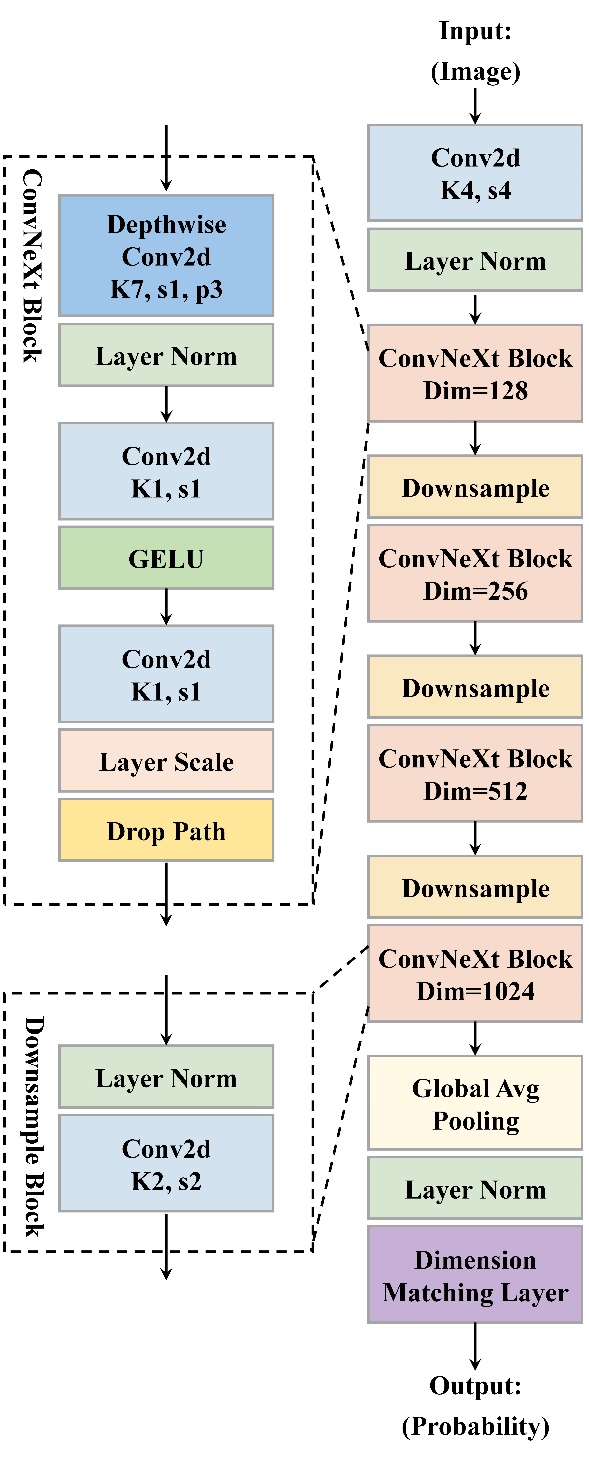


**Fig. S1. Detailed network architecture of deep learning models**

The neural network is composed of many different types of layers and the layer is also the basic unit of forward calculation. In Fig. S1, the neural network includes some convolutional layers, depth-wise convolution layers, maximum polling layers, layer normalization layers, fully connected layers, activation function layers, drop-paths, and one global average layer.

**Table S1. Detailed hyperparameter configuration of the deep learning models**

| **Layer name** | **Output size** | **Description** |
| --- | --- | --- |
| Block_1 | 128×117×70 | $\left[ \begin{matrix} 7\times7, & 128 \\ 1\times1, & 128 \\ 1\times1, & 128 \end{matrix} \right]$x 3 |
| Block_2 | 256×58×35 | $\left[ \begin{matrix} 7\times7, & 128 \\ 1\times1, & 128 \\ 1\times1, & 256 \end{matrix} \right]$x 3 |
| Block_3 | 512×29×17 | $\left[ \begin{matrix} 7\times7, & 256 \\ 1\times1, & 256 \\ 1\times1, & 512 \end{matrix} \right]$x 27 |
| Block_4 | 1024×14×8 | $\left[ \begin{matrix} 7\times7, & 512 \\ 1\times1, & 512 \\ 1\times1, & 1024 \end{matrix} \right]$x 3 |
| GAP | 1024x1 | 1024-d feature vector |
| FC | 1x1 | 2-d feature vector |

In this study, Deep Learning Model (DLM-1, DLM-2) are mainly based on ConvNeXt . We only made a few modifications in the fully connected layer to achieve two-category classification tasks. The detailed structure of the network is shown in Additional File: Table S1.
